# Photo Quiz: Hidden travelers and visible marks—about two imported dermatological cases in Grenoble, France

**DOI:** 10.1128/jcm.01144-25

**Published:** 2025-11-12

**Authors:** Paul A. Bertoye, Foucauld Guinamard, Marie Gladys Robert, Caroline Henry, Florence Tourneur, Marie-Pierre Brenier-Pinchart, Truong-Giang Nguyen

**Affiliations:** 1 Department of Infectious & Tropical Diseases, University of Grenoble Alpes, CHU Grenoble Alpes 36724 , Grenoble, France; 2 Laboratory of Parasitology & Mycology, University of Grenoble Alpes, CHU Grenoble Alpes 36724 , Grenoble, France; 3 Department of Pediatric Infectious & Tropical Diseases, University of Grenoble Alpes, CHU Grenoble Alpes 36724 , Grenoble, France; 4 Institute for Advanced Biosciences (IAB), INSERM-CNRS, Grenoble Alpes University, Grenoble, France; 5 Department of Infectious & Tropical Diseases, University of Grenoble Alpes, CHU Grenoble Alpes-Voiron, Voiron, France; 6 Pediatrician, Saint-Ismier, France; Mayo Clinic Minnesota, Rochester, Minnesota, USA

**Keywords:** dermatology, nodule

## PHOTO QUIZ 

## PATIENT 1

**Fig 1:**
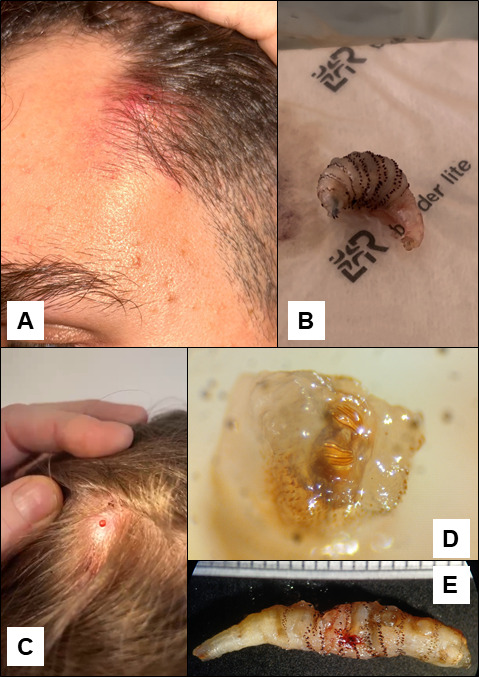
Clinical and parasitological features of our 2 cases; (**A**) cutaneous lesion of patient 1, (**B**) larva extracted from patient 1, (**C**) cutaneous lesion of patient 2, (**D**) respiratory spiracles of the larva extracted from patient 2, (**E**) larva extracted from patient 2.

A 23-year-old male with no known chronic diseases or current medications presented with a painful, inflammatory nodule on the left temporal region (Fig. 1A). The patient also exhibited ipsilateral cervical lymphadenopathy, with symptom onset occurring 10 days following his return from a 10-day period in Belize, during which he reported frequent excursions into jungle environments. After initial care in Voiron (France), antibiotic therapy with pristinamycin was started, prescribed for a presumed furuncle, and yielded no clinical improvement. Subsequently, empiric systemic antibiotic treatment with amoxicillin-clavulanate was initiated for a 7-day course. This regimen resulted in a temporary amelioration of inflammatory signs. However, shortly after completion of the antibiotic treatment, the lesion began to exude serosanguinous drainage with relapse of a periorbital edema.

A surgical layout of the lesion did not allow for the removal of any pus or larvae. Occlusive dressing was put in place, and the nurse changing the dressing detected a larva that was manually extracted 2 days later from the lesion (Fig. 1B).

## PATIENT 2

An 8-year-old female with no significant prior medical history presented to her attending pediatrician in Grenoble (France) with a pruritic nodule of the scalp, accompanied by intermittent serosanguinous exudation (Fig. 1C). The symptoms began 1 month after a 15-day trip to Cayenne and Montjoly, French Guiana. After failure of antibiotic therapy with amoxicillin-clavulanate, clinical examination revealed the nodule exhibited regular, rhythmic movement, described as appearing to be “breathing” by the parents of the young girl.

Instrumental traction of the content of the nodule allowed for a larva to be removed.

In these two cases, an approximately 20 mm long larva (Fig. 1B and E), presenting visible cuticular spines, was extracted either instrumentally or manually.

What is your diagnosis?

## ANSWER TO THE PHOTO QUIZ

Microscopic examination of the larvae posterior spiracles revealed three sinuous slits lacking a discernible peritreme (Fig. 1D), and general examination showed coarse cuticular spines oriented posteriorly across all but three body segments, enabling identification as third-instar *Dermatobia hominis* larvae (1).

Furuncular myiasis due to *D. hominis* is endemic in Central and South America. Although many cases have been reported from travelers or workers from South America, the majority of French cases are diagnosed in French Guiana. Gravid female *D. hominis* exhibit phoretic behavior, ovipositing eggs onto hematophagous insect vectors, typically mosquitoes. Following a 4–9-day incubation period, first-instar larvae penetrate the human skin during mosquito blood-feeding. The larva develops subsequently into a third-instar larva, forming a cutaneous nodule over 6–12 weeks. Ultimately, the larva breaches the epidermis to facilitate respiration via its posterior spiracles, resulting in the characteristic clinical presentation of furuncular myiasis (2).

Diagnosis is often clinical, based on visualizing larval respiration through the skin, or with dermoscopic examination (3). Symptoms may include a painful, pruritic, and sometimes inflammatory cutaneous nodule with serosanguinous exudation.

Treatment involves mechanical or surgical excision of larger larvae. Smaller larvae can be suffocated using occlusive agents like petroleum jelly or cyanoacrylate before mechanical extraction (4). Species identification relies on morphological features: macroscopic examination of extracted larvae allows identification by the general presentation of the larvae itself (size, shape, and distribution of cuticular spines); more precise identification is often achieved by examining posterior spiracles, either by binocular examination or after removal of the spiracles and microscopic examination. The number and shape of the spiracles, as well as the presence or absence of a peritreme, help distinguish *D. hominis* from other agents of furunculoid myiases.

While not life-threatening, early diagnosis of furuncular myiasis is essential for optimal lesion care, minimizing the risk of secondary infection—especially in the pediatric population, where this complication has been reported more frequently than in adults (5, 6)—as well as reducing unaesthetic scarring and misdiagnosis. These cases highlight that, despite the rarity of this diagnosis outside Central and South America, imported cases can present even after short stays in endemic zones.
